# New-onset acute psychosis as a manifestation of lupus cerebritis following concomitant COVID-19 infection and vaccination: a rare case report

**DOI:** 10.1186/s12888-023-04924-4

**Published:** 2023-06-12

**Authors:** Feten Fekih-Romdhane, Farah Ghrissi, Souheil Hallit, Majda Cheour

**Affiliations:** 1 Faculty of Medicine of Tunis, Tunis Al Manar University, Tunis, Tunisia; 2 The Tunisian Center of Early Intervention in Psychosis, Department of Psychiatry “Ibn Omrane”, Razi Hospital, Manouba, 2010 Tunisia; 3 School of Medicine and Medical Sciences, Holy Spirit University of Kaslik (USEK), P.O. Box 446, Jounieh, Lebanon; 4 Applied Science Research Center, Applied Science Private University, Amman, Jordan; 5 Research Department, Psychiatric Hospital of the Cross, Jal Eddib, Lebanon

**Keywords:** Systemic lupus erythematosus, Acute psychosis, Lupus cerebritis, COVID-19, Vaccination

## Abstract

**Background:**

Rare cases of COVID-19 infection- and vaccine-triggered autoimmune diseases have been separately reported in the literature. In this paper, we report the first and unique case of new onset acute psychosis as a manifestation of lupus cerebritis following concomitant COVID-19 infection and vaccination in a previously healthy 26-year-old Tunisian female.

**Case presentation:**

A 26-years old female with a family history of a mother diagnosed with schizophrenia, and no personal medical or psychiatric history, was diagnosed with mild COVID-19 infection four days after receiving the second dose of Pfizer-BioNTech COVID-19 vaccine. One month after receiving the vaccine, she presented to the psychiatric emergency department with acute psychomotor agitation, incoherent speech and total insomnia evolving for five days. She was firstly diagnosed with a brief psychotic disorder according to the DSM-5, and was prescribed risperidone (2 mg/day). On the seventh day of admission, she reported the onset of severe asthenia with dysphagia. Physical examination found fever, tachycardia, and multiple mouth ulcers. Neurological evaluation revealed a dysarthria with left hemiparesis. On laboratory tests, she had severe acute kidney failure, proteinuria, high CRP values, and pancytopenia. Immune tests identified the presence of antinuclear antibodies. Brain magnetic resonance imaging (MRI) revealed hyperintense signals in the left fronto-parietal lobes and the cerebellum. The patient was diagnosed with systemic lupus erythematosus (SLE) and put on anti-SLE drugs and antipsychotics, with a favorable evolution.

**Conclusions:**

The chronological relationship between COVID-19 infection, vaccination and the first lupus cerebritis manifestations is highly suggestive, albeit with no certainty, of the potential causal link. We suggest that precautionary measures should be taken to decrease the risk of SLE onset or exacerbation after COVID-19 vaccination, including a systematic COVID-19 testing before vaccination in individuals with specific predisposition.

## Introduction

So far, the coronavirus disease 19 (COVID-19) pandemic has caused substantial morbidity and mortality rates around the globe. While most often presenting as flu-like symptoms, other severe manifestations and complications underpinned by the cytokine release storm (CRS) may occur. This engenders a marked upsurge of systemic inflammation and an acute severe immune-response, which may in turn lead to the exacerbation or even emergence of autoimmune disorders [1]. In an attempt to create an immune barrier among population and attenuate the spread of the virus, various vaccines have been timely developed and have been shown to be safe and effective in the majority of populations vaccinated. Nevertheless, rare adverse effects have recently been reported, including the triggering of autoimmune diseases [2]. Therefore, autoimmune diseases induced by both infection and vaccination have become a serious concern.

A challenging autoimmune disease is systemic lupus erythematosus (SLE). One hypothesized etiology for the development of SLE is viral [3]. Viral agents (e.g., Epstein–Barr virus, parvovirus B19, retroviruses and cytomegalovirus) have been suggested to trigger immune reactions against the self-antigens, thus leading to autoimmunity [3]. In this regard, some authors have also reported cases of SLE manifesting after COVID-19 infection in a 39-year-old Iranian/Persian man [4], a 32-year-old Kenyan woman [5], an 85-year-old Italian woman [6], and a 38 year old Iranian woman [7]. Both SLE and COVID-19 are characterized by a complex clinical manifestation; and share similar disease characteristics including multi-organ complications (e.g., arthralgia, cytopenia, hemophagocytic lymphohistiocytosis, interstitial pneumonia, and myocarditis) [8]. In addition, both diseases affect the Central Nervous System and may manifest as neuropsychiatric symptoms (known as lupus cerebritis and neuro-COVID); with psychosis being amongst the least uncommon and most dreaded neuropsychiatric manifestations of SLE [9] and COVID-19 [10]. Herein, we report the first and unique case of new onset acute psychosis as a manifestation of lupus cerebritis following both COVID-19 infection and vaccination in a previously healthy 26-year-old Tunisian female. We then discuss possible mechanisms linking these two entities and potential therapeutic approaches based on the literature.

## Written consent for publication from the patient

Informed written consent was obtained from the patient for the use and publication of the clinical data and results from clinical examinations for research.

## Case presentation

A 26-years old female with a family history of a mother diagnosed with schizophrenia, and no personal medical or psychiatric history, was diagnosed with mild COVID-19 infection by nasopharyngeal and throat swab reverse transcription-polymerase chain reaction (RT-PCR), four days after receiving the second dose of Pfizer-BioNTech COVID-19 vaccine. She was asymptomatic when she received the COVID-19 vaccine. Two weeks later, she presented to the emergency department with chest pain, where physical examination revealed a heart rate of 110/min. A cardiac ultrasound examination showed a pericardial effusion suggestive of pericarditis. She was started on aspirin and colchicine, with a slow recovery.

One month after receiving the vaccine, she presented to the psychiatric emergency department with acute psychomotor agitation, incoherent speech and total insomnia evolving for five days. She had no recent history of trauma, and no lifetime history of substance use. Routine investigations were done to rule out a differential diagnosis of psychosis according to first signs and the clinical examination, such as cerebrovascular (e.g., stroke, subdural hematomas, cerebral tumors), seizure (e.g., partial complex seizures, temporal lobe epilepsy), metabolic (e.g., phaeochromocytoma), infectious (neurosyphilis, HIV), and endocrine (dysthyroïdism) disorders [11]. Precisely, laboratory tests (including thyroid blood tests and syphilis/HIV serologies) showed an isolated mild anemia. Her electroencephalographic (EEG) and CT brain-scan found no abnormalities. She, and her family members, denied any substance use. She was admitted to our psychiatric unit, where psychiatric examination revealed severe anxiety, delusions of persecution and reference, auditory hallucinations, disorganized speech and behavior. The patient had no delirium symptoms and a lack of insight. Her neurological examination was normal. She was started on intramuscular haloperidol 10 mg/ day and diazepam 10 mg/day. There was a complete resolution of psychotic symptoms within four days of treatment initiation. She was thus diagnosed with a brief psychotic disorder according to the DSM-5 [12], and was prescribed risperidone (2 mg/day).

**Fig. 1:**
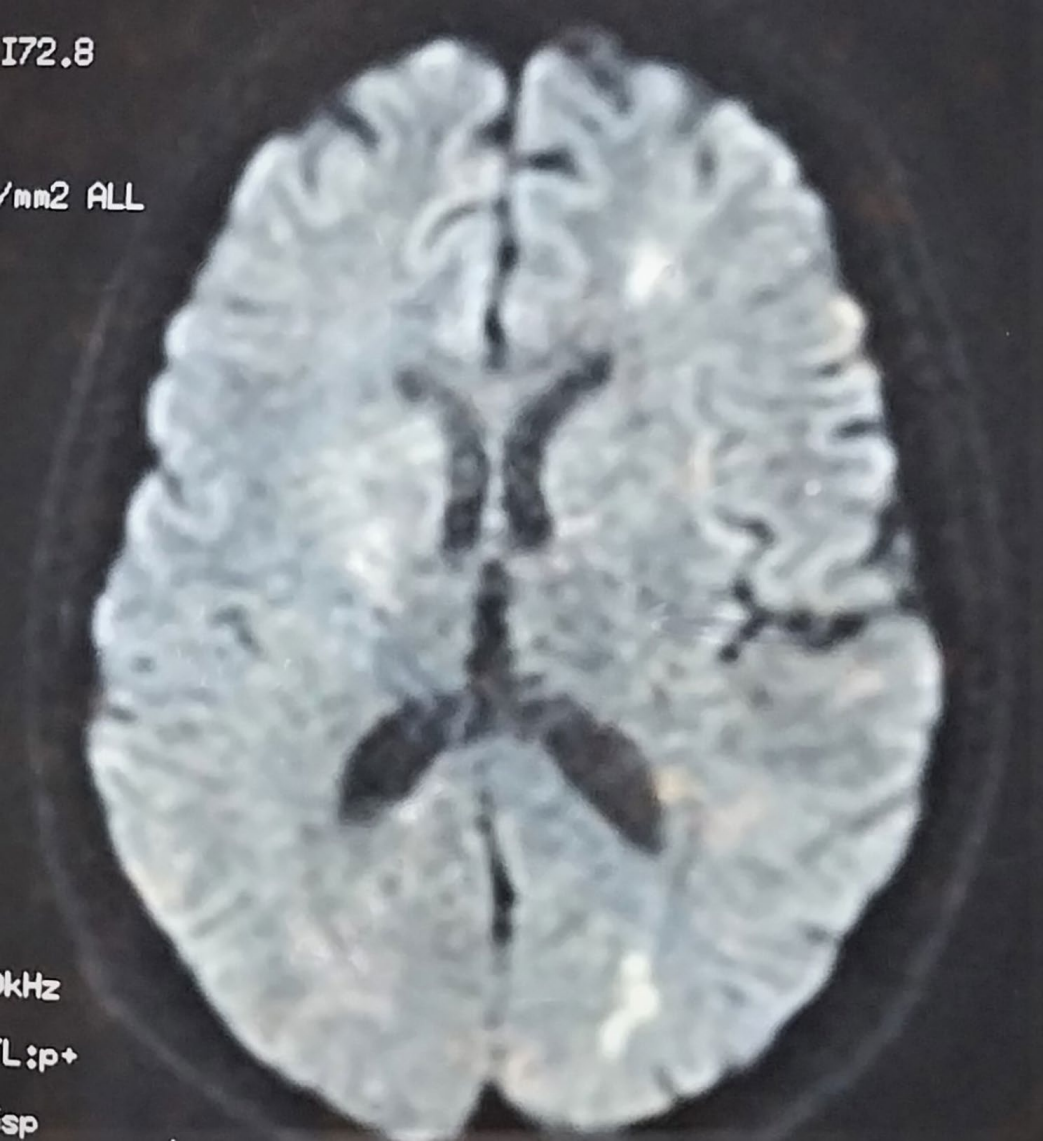
Magnetic resonance imaging (MRI) shows multifocal Hyperintense lesions of the brain (in the left fronto-parietal lobes and the cerebellum) on T2 weighted-images

On the seventh day of admission, while her discharge has been planned and discussed with the family, she reported the onset of severe asthenia with dysphagia. Physical examination found fever (body temperature of 40 degrees Celsius), tachycardia (heart rate of 123 beats/min), and multiple mouth ulcers. Thoracic auscultation found normal lung and heart sound. A second neurological evaluation revealed a dysarthria with left hemiparesis, evoking an acute stroke. Another psychiatric examination found a concomitant relapse of psychiatric symptoms. On laboratory tests, she had severe acute kidney failure, proteinuria, high CRP values, and pancytopenia. Infectious investigation was negative. The SARS-CoV-2 PCR with nasopharyngeal swab was negative. Chest X-ray showed bilateral pleural effusion. Immune tests identified the presence of antinuclear antibodies. The anti-ENA (extractable nuclear antigen) antibody screen showed strongly positive anti-SSA, anti-Ro52, anti-SSB, as well as weakly positive anti-nucleosome, and anti-histone antibodies. Brain magnetic resonance imaging (MRI) revealed hyperintense signals in the left fronto-parietal lobes and the cerebellum (Fig. 1). Based on these clinical and laboratory evidence, the patient was diagnosed with SLE according to the 2019 American College of Rheumatology/European League Against Rheumatism (ACR/EULAR) classification criteria [13]. She was then transferred to the internal medicine department and put on Hydroxychloroquine (400 mg daily), prednisone (40 mg daily), Cyclophosphamide (1 g monthly) and risperidone (2 mg daily). Both the SLE and psychotic symptoms improved within one month, and she was discharged. During outpatient follow-up, she was free from neurological and psychiatric symptoms and had good tolerance to treatment.

## Discussion

In this paper, we describe to our knowledge the first case of lupus cerebritis manifesting as new-onset acute psychosis 3 to 4 weeks following both COVID-19 infection and vaccination in a young female adult with no previous medical or psychiatric history. It is worth mentioning that the patient had one episode of acute pericarditis around two weeks after the COVID-19 infection, and two weeks prior to the onset of the acute psychosis. She also had an acute stroke. Both pericarditis and stroke can either be due to the COVID-19 [14, 15], or to SLE [16, 17]. We could find only one case report of lupus cerebritis occurring three weeks after COVID-19 infection in a 29-year-old female with a past medical history of SLE, and which manifested as slow speech, psychomotor agitation, and intermittent choreiform movements in the upper part of the body [18]. The existing new-onset SLE cases reported in the literature developed either concurrently with COVID-19 infection, or within one to six weeks after infection/vaccination (see Table 1).

**Table 1:** Adult patients with new-onset SLE triggered by COVID-19 infection or vaccination

Authors, Year, Reference	Country	Patients characteristics	Chronological link to COVID-19 infection/ vaccination	Clinical presentation	Investigations	Diagnosis	Treatment and evolution
**New-onset SLE triggered by COVID-19 infection (n = 7)**							
Ramachandran et al., 2022 [29],	Boston, Massachusetts	A 53-year-old male with a past medical history of essential hypertension and chronic kidney disease 3	One month following COVID-19 infection	Persistent fatigue, malaise, wrist pain, intermittent nausea and vomiting, exertional dyspnea, bilateral lower extremity swelling and decreased urine output.	*Blood/urine tests: elevated creatinine, nephrotic-range proteinuria *Serologic tests: elevated ANA and anti- dsDNA, decreased C3 and C4 levels *Chest X-ray: bilateral pleural effusions and vascular congestion *Renal biopsy : focal segmental glomerulosclerosis.	SLE with stage IV lupus nephritis	*Treatment: plasmapheresis and immunosuppressants. *Evolution: significant improvement in his symptoms.
Hali et al., 2021 [30],	Morocco	A 25-year-old female patient, with no particular medical history	Current COVID-19 infection	Rash with diffuse myalgias, asthenia, fever, a diffuse maculopapular exanthema with palmarplantar involvement, periorbital edema, infiltrated purpuric lesions of the lower limbs, with multiple labial and palatal erosions.	*Chest CT scan: bilateral pulmonary infiltrates of probable viral origin *PCR test: positive for SARS-CoV-2 RNA *Blood tests: aregenerative normocytic normochromic anemia, leukopenia, neutropenia, and lymphopenia * Serological tests: positive ANA and anti- dsDNA, low complement levels, negative antiphospholipid antibodies, and positive proteinuria *Cardiac ultrasound: stage 3 mitral insufficiency without pericardial effusion *Skin biopsy : leukocytoclastic vasculitis	SLE with leukocytoclastic vasculitis, combined with COVID-19	*Treatment: steroid therapy with methylprednisolone along with the usual treatment for COVID-19. *Evolution: clinical and biological improvement.
Gracia-Ramos et al., 2021 [31],	Mexico	A 45-year-old man	Current COVID-19 infection	Respiratory symptoms, bilateral pleural effusion, ascites, splenomegaly, severe thrombocytopenia and renal failure with proteinuria and hematuria	*Blood tests: Hyperazotemia, hypoalbuminemia, thrombocytopenia, mild anemia, prolonged activated partial thromboplastin time and elevated fibrinogen *Chest X-ray: bilateral reticular pattern with vascular enlargement *Abdominal ultrasonography: small bilateral pleural effusion, peritoneal fluid and splenomegaly. * PCR: positive * Immunologic test : ANA positive with coarse speckled pattern	SLE combined with COVID-19	*Treatment: Glucocorticoid pulses *Evolution: renal function improved. However, thrombocytopenia was refractory to IV immunoglobulin and rituximab, so the patient underwent splenectomy
Mantovani Cardoso et al., 2020 [32],	USA	A 18-year-old female with a past medical history of autism spectrum disorder and panic disorder	Current COVID-19 infection	Hemodynamic collapse and respiratory failure, progressed to cardiac arrest, and pericardial tamponade. Then, severe acute respiratory distress syndrome, severe ventricular dysfunction, and worsening renal function with proteinuria and hematuria, multiple deep venous thrombosis	* Chest X-ray: bilateral pleural effusions * Immunologic tests: positive ANA and anti- dsDNA, lupus anticoagulant, and anticardiolipin B. C3 and C4 levels were low. * SARS-Cov-2 PCR : positive after 2 negative tests.	SLE combined with COVID-19	*Treatment: pulse steroids, ceftazidime, vancomycin, azithromycin, hydroxychloroquine, one session of plasmapheresis; then 1 dose of tocilizumab. *Evolution: She went into pulseless electrical activity arrest on day 17 of hospitalization and passed away
Slimani et al., 2021 [33],	Morocco	A 23-year‐old female, with no past medical history	Current COVID-19 infection	fever, fatigue, dry cough and dyspnea.	*Blood tests: lymphopenia, elevated prothrombin time and activated partial- thromboplastin time, elevated D‐dimer *PCR test: positive for SARS-CoV‐2 *Chest computed tomography scan: bilateral pulmonary infiltrates * Serologic testing: presence of ANA, anti- dsDNA, anticardiolipin IgG and IgM antibodies, anti‐β2‐glycoprotein I IgG, IgA antibodies, and lupus anticoagulant	SLE with anti-phospholipid antibody syndrome, combined with COVID-19	*Treatment: single dose of chloroquine, that was discontinued because of cardiac toxicity *Evolution: invasive mechanical ventilation initiated for hypoxemic respiratory failure; skin rash on the trunk appeared; then the patient died 16 days after the diagnosis of COVID-19 was made
Zamani et al., 2021 [4],	Iran	A 39-year-old man	Two months following COVID-19 infection	Fever, scaling on the palms of the hands and feet, lower extremity edema, ankle swelling, and a weight loss of about 15 kg, anorexia, and headache during 2 months.	*PBS: toxic granulation *Urine analysis: 2 + proteinuria *EMG and NCV: motor and sensory polyneuropathies * Serologic testing: elevated anti-La/SSB and anti-SSA/Ro, anti-dsDNA, and anti-CCP antibodies	SLE	*Treatment: pulse methylprednisolone; then hydroxychloroquine, prednisolone cyclophosphamide, gabapentin, and vitamin B *Evolution: clinical and biological improvement.
Bonometti et al., 2020 [6],	Italy	An 85-year-old woman	A previous (unknown date) COVID-19 infection	Unconsciousness, hemodynamic instability with severe hypotension and diffuse marbled.	*Blood tests: Neutrophilic leukocytosis, lymphopenia, elevation of C-reactive protein, thrombocytopenia, severe acute kidney injury with hypokalemia, hypernatremia and elevation of ferritin and LDH *Chest X-ray: accentuation of the lung design at the basis and pleural effusion. *Nasal swab for COVID-19: negative; IgG for COVID-19 positive (IgM negative) * Serologic testing: positivity for ANA	SLE	*Treatment: hydroxychloroquine in association to steroid therapy *Evolution: not described
**New-onset SLE triggered by COVID-19 vaccination (n = 14)**							
Liu and Messenger, 2021 [34],	USA	An 70-year-old man with a history of lung cancer	2.5 months following COVID-19 vaccine type Pfizer-BioNTech (2nd dose)	Violaceus plaques with central hypopigmentation surrounding erythema, and peripheral scaling on the abdomen, back and bilateral upper extremities	*Serologic tests: ANA, anti-Ro/SS-A	SLE (Subacute cutaneous lupus erythematosus)	*Treatment: Glucocorticoid *Evolution : improvement after 1 month ; then lost to follow-up
Patil & Patil, 2021 [35],	India	A 22-year-old female with a history of infective jaundice (non-B) at the age of 9 years	25 days following COVID-19 vaccination with Covishield	Polyarthralgia (small as well as large joints), bipedal edema, cutaneous rash over fingertips, and petechiae over lower limb	* Neck and abdominal ultrasound examination: bilateral cervical lymphadenopathy, (Level 1) and mild hepatomegaly, respectively. *Serological tests: ANA positive for dsDNA, nucleosomes, histones, and AMA m2. Anti-nuclear antibodies strongly positive. *Blood tests: anemia, thrombocytopenia, DCT weakly positive. *Urine routine: 1 + albuminuria and 3–4 RBC per high power field.	SLE with anemia of chronic diseases	*Treatment: prednisolone (50 mg daily), hydroxychloroquine (400 mg daily), mycophenolate mofetil (2 g daily), furosemide (20 mg daily), telmisartan (20 mg daily), folic acid, calcium, and vitamin D3. *Evolution: Follow-up after a month showed significant improvement.
Garmonal et al., 2022 [36],	Brazil	A 27-year-old woman	3 weeks following COVID-19 vaccine type Astra-Zeneca (2nd dose)	Itchy and painful skin lesions, oral mucosa, fever, fatigue, and later alopecia (after 15 days)	*Blood tests: anemia, leucopenia, lymphopenia, altered urine protein/creatinine ratio * COVID-19 RT-PCR: negative *Serologic tests: ANA, anti-Sm, anti-Ro/SS-A, anti-La/SS-B, anti-dsDNA, low C3 and C4	SLE and alopecia areata	*Treatment: Prednisone 80 mg/day, hydroxychloroquine 400 mg/day *Evolution: not described
Kaur et al., 2022 [37]	USA	A 54-year-old Asian man with a history of stable Sjogren’s syndrome	2 weeks following COVID-19 vaccine type Pfizer-BioNTech (2nd dose)	Fever, fatigue, generalized malaise, loss of appetite, unintentional weight loss, chest heaviness, shortness of breath, worsening of dry mouth and eyes, and burning and pain on bilateral feet. After around a week, he developed confusion and recent memory amnesia.	*Blood tests: pancytopenia, hyponatremia, hypochloremia, elevated liver function tests, and significant hypocomplementemia * COVID-19 RT-PCR: negative *Serologic tests: ANA, anti-dsDNA, anti-Ro/ SS-A, anti-La/SS-B, low C3 and C4	SLE	*Treatment: Prednisone 60 mg/day and mycophenolate mofetil 1,000 mg/day *Evolution: after 2 weeks of treatment, improvement of the neuropsychiatric manifestations.
Molina-Rios et al., 2022 [38],	Colombia	A 42-year-old woman with a history of two spontaneous abortions	2 weeks following COVID-19 vaccine type Pfizer-BioNTech (1st dose)	Polyarthralgia, dyspnea, pulmonary thromboembolism (APS), cardiac tamponade	*Blood tests: high C-reactive protein (CRP) level, elevated D-dimer levels *Serologic tests: ANA, anti-dsDNA, low C4, anti-beta-2-glycoprotein IgG 90 U/mL and IgM 46 U/mL *Computed tomography (CT) pulmonary angiogram: pulmonary embolism	SLE and secondary antiphospholipid syndrome (APS)	*Treatment: Hydroxychloroquine 200 mg/day, intravenous methylprednisolone 250 mg/day for 3 days, followed by prednisolone 0.5 mg/kg/ day, azathioprine 100 mg/ day, anticoagulation *Evolution: clinical and biological improvement.
Zavala- Miranda et al., 2021 [39],	Mexico	A 23-year-old woman with no previous medical history of disease	One weeks following COVID-19 vaccine type Astra-Zeneca (1st dose)	Abrupt eyelid edema, foamy urine that progressed to anasarca, hair loss	*Blood tests: Lymphopenia *proteinuria *Serologic tests: ANA, anti-dsDNA, antibodies and low C3 and C4 levels *kidney biopsy : secondary membranous nephropathy, with diffuse thickening of the basement glomerular membrane and mild mesangial expansion	SLE with class V lupus nephritis	*Treatment: Prednisone, hydroxychloroquine, mycophenolate mofetil *Evolution: clinical and biological improvement.
Hidaka et al., 2022 [40],	Japan	A 53-year-old woman with a medical histories of bronchial asthma, Vogt–Koyanagi–Harada disease, and Hashimoto disease	2 weeks following COVID-19 vaccine type Pfizer-BioNTech (1st dose)	Evans’ syndrome	*Blood tests: thrombocytopenia with mild anemia, elevated indirect bilirubin, elevated lactate dehydrogenase, and low haptoglobin *Serologic tests: ANA, lupus anticoagulant, low C3 and C4, Coombs test	SLE associated with Evans syndrome and exacerbation of bronchial asthma	*Treatment: Prednisone 60 mg/day *Evolution: clinical and biological improvement.
Nune et al., 2021 [41],	United Kingdom	A 24-year-old man	2 weeks following COVID-19 vaccine type Pfizer-BioNTech (2nd dose)	Polyarthralgia, polyarthritis, joint stiffness, fever and fatigue	*Blood tests: leucopenia, lymphopenia *Serologic tests: ANA, anti-dsDNA, low C3 and C4 * COVID-19 RT-PCR: negative	SLE	*Treatment: Prednisone 60 mg/day, methotrexate 15 mg/week *Evolution: clinical and biological improvement.
Kim et al., 2022 [42],	Korea	A 60-year-old woman with a history of a skin rash in 2015	2 months following COVID-19 vaccine type Pfizer-BioNTech (2nd dose)	Asthenia, fever, anemia, pitting edema	*Blood tests: Proteinuria, thrombocytopenia, lymphopenia, hematuria, increased creatinine levels, * kidney biopsy: class III lupus nephritis *Serologic tests: ANA, anti-dsDNA, anti-Sm, low C3 and C4	SLE with stage III lupus nephritis	*Treatment: Prednisone, glucocorticoid pulse, cyclophosphamide, hydroxychloroquine 200 mg/day *Evolution: clinical and biological improvement.
Sogbe et al., 2023 [43],	Spain	A 72-year-old female with a history of kidney transplantation in 2004 due to a chronic kidney failure secondary to membranoproliferative glomerulonephritis; then chronic hemodialysis since 2017 for renal graft dysfunction	One week after COVID-19 Pfizer BioNTech vaccine (3rd dose)	Pleuritic chest pain	* PCR test: negative *Serological tests: ANA positive, anti-dsDNA, and anti-histone antibodies positive with low serum C3 level *PET/CT: a focal myocardial and pericardial inflammatory process in the cardiac apex	SLE with myopericarditis	*Treatment: oral prednisone (1 mg/kg) and beta-blockers *Evolution: clinical and biological improvement.
Sakai et al., 2023 [44],	Japan	A 26-year-old woman	3–4 weeks after COVID-19 Pfizer/BioNTech vaccination (the two doses were received with a 3-week interval)	Eyelid oedema, bilateral leg oedema, and weight gain of 5 kg	*Blood/urine tests: thrombocytopenia, hypocomplementemia, decreased serum protein and increased urinary protein levels. *Serological tests: ANA, anti-dsDNA, anticardiolipin, and anti-SS-A antibodies positive	SLE with lupus nephritis Class II	* Treatment: methylprednisolone pulse therapy, post-prednisolone therapy (1 mg/kg/day, tapering off), hydroxychloroquine 200 mg/day, mycophenolate mofetil 1000–2000 mg/day, and belimumab 200 mg/week *Evolution: Clinical remission.
		A 62-year-old man with a history of cerebellar infarction and ablation treatment for atrial fibrillation (7 years ago), and right putaminal haemorrhage with surgical treatment (4 years ago)	1–4 weeks after COVID-19 Pfizer/BioNTech vaccination (the two doses were received with a 3-week interval)	Fever, weakness in the upper and lower limbs. Three weeks later, he fell, with a decline in his cognitive function	*Blood tests: pancytopenia *Serological tests : hypocomplementemia, ANA and anti-dsDNA antibody positivity	SLE	* Treatment: Methylprednisolone pulse with post-prednisolone therapy (1 mg/kg/day, tapering off) and intravenous cyclophosphamide 750 mg/day *Evolution: Clinical remission.
Beynon et al., 2022 [45],	United Kingdom	A 29-year-old female with no prior medical history	One week following COVID-19 Pfizer/BioNTech vaccination	Widespread pruritus, fatigue, myalgia, arthralgia, fever and night sweats, polyarticular inflammatory arthritis, oral ulceration, Raynaud’s, pleuritic chest pain, palmar purpuric rash, and a widespread tender urticarial rash.	*Blood tests: pancytopenia, low complement C3/C4, *Serological tests: positive anti-dsDNA, anti-Ro antibody, anti-La antibody, weakly positive anti-RNP and anti-C1q antibodies * PET/CT: widespread lymphadenopathy *Skin biopsy : lupus vasculitis	SLE with urticarial vasculitis	* Treatment: Hydroxychloroquine + Prednisolone (60 mg). *Evolution: the patient developed a class 3 lupus nephritis. She was pulsed with 500 mg IV methyl prednisolone over 3 days and commenced mycophenolate 1 g BD. Within weeks she was in clinical remission.
		A 70-year-old female with a history of diverticulosis, uterine fibroids and small hand joint osteoarthritis	8 days following the first dose of the COVID-19 Oxford-AstraZeneca vaccination	Six-week history of bilateral symmetrical small and large joint synovitis.	*Blood tests: lymphopenia, raised CRP and ESR. *Immunological assessment: ANA were weakly positive with a homogenous pattern. DsDNA was raised and C4 reduced.		*Treatment: A short reducing course of oral prednisolone. *Evolution: Symptoms improved, with no recurrence on stopping steroids. She has continued elevation in DsDNA, which has led to the adoption of a conservative management approach

CT: computed tomography; PCR: polymerase chain reaction; ANA: antinuclear antibody; anti- dsDNA: anti‐double stranded DNA antibodies; Ig; immunoglobulin; PBS: peripheral blood smear; EMG: Electromyography; NCV: nerve conduction velocity; anti-CCP: anti-cyclic citrullinated peptides; DCT: Direct Coombs test; PET/CT: Positron Emission Tomography/computed tomography; ESR: Erythrocyte sedimentation rate; SS-A: Sjogren’s syndrome type A ¨.

Acute psychosis as the first and main presenting manifestation of SLE is rarely encountered in clinical practice [19]. The etiopathogenesis of psychosis due to neuro-lupus is multifactorial, yet complex and unclear [20]. The two main, and likely complementary proposed mechanisms are (1) autoimmune/ inflammatory and (2) ischemic or thrombotic pathways [20, 21]. The autoimmune-mediated neuro-inflammatory pathway involves an increased permeability of the blood-brain barrier, with neuronal autoantibodies intrathecal migration and intracranial generation of pro-inflammatory mediators (cytokines and others) [20]. As for the ischemic pathway, it originates in cerebral micro-angiopathy, which is mediated by immune complexes, antiphospholipid (aPL) antibodies, and complement activation [20].

Although psychosis has been widely reported as a lupus-related phenomenon and as a well-established manifestation of lupus cerebritis, the association between psychosis and SLE in the present case deserves to be deeply discussed. Some hypotheses seem plausible. The first hypothesis is a possible direct role of viral infection on the concomitant but fortuitous occurrence of SLE [3] and psychosis [22] in a genetically loaded young adult (i.e. family history of schizophrenia). However, it is difficult and premature to conclude whether a direct and causal link exists between COVID-19, SLE and psychosis, given that both psychosis and SLE have been reported in individuals who had or had not a prior history of these diseases prior to contracting COVID-19. The second and most plausible hypothesis is that the patient would have developed a lupus-related psychosis [9], as a severe manifestation of COVID-19 vaccination in an infected individual. In this case, the COVID-19 vaccination coupled with a concomitant infection would have precipitated a strong immune dysregulation and the subsequent onset of a severe form of SLE. This hypothesis is supported by the fact that both COVID-19 infection and vaccine have been shown to trigger SLE [1, 2]. Several explaining mechanisms have been suggested. COVID-19 infection would incite autoimmunity through molecular mimicry between human proteins and the virus [23]. In a similar way, the vaccine produces antibodies against COVID-19 spike protein that can cross-react with antigens from the host and trigger autoimmune diseases in predisposed individuals [24]. A few cases of individuals who developed de novo SLE after receiving Pfizer-BioNTech COVID-19 mRNA vaccine have been published in the literature (e.g., a 68-year-old Caucasian woman [25], a 42-year-old woman [26], a 24-year-old Israeli male [27], a 27-year-old Puerto Rican woman [28]); however, we could find no reports on the onset of SLE after both vaccine and infection. In our patient, the chronological relationship between COVID-19 infection, vaccination and the first lupus cerebritis manifestations is highly suggestive, albeit with no certainty, of the potential causal link. In addition, the fact that our patient developed SLE symptoms after receiving the second dose of the vaccine suggests that the vaccine-induced immune response was further enhanced by the covid-19 infection, and that the combined effect of both triggers could explain the development of SLE.

People with SLE has proven to have the worse outcomes from COVID-19, and have thus been prioritized to receive the vaccine [24]. The international vaccination against COVID in systemic lupus (VACOLUP) initiative stipulated that, despite the risk of flares, COVID-19 immunization was rather well accepted in patients with SLE [24]. Therefore, we emphasize that our report should not discourage against vaccination, albeit vigilance should be raised among susceptible population about the possible emergence of SLE after mRNA vaccination. Therefore, we suggest that precautionary measures should be taken to decrease the risk of SLE onset or exacerbation after COVID-19 vaccination, including a routine COVID-19 testing before vaccination in individuals with specific predisposition (e.g., a family history of either psychosis or autoimmune diseases).

Given its wide array of clinical manifestations, the diagnosis of SLE may be particularly challenging, especially when lupus cerebritis is the initial presenting feature. Primary psychosis manifestations of SLE may mislead or delay the diagnosis and thus delay appropriate management with poor outcomes and significant impact on mortality. Antipsychotic medication needs to be prescribed in severe psychosis along with required treatment for SLE. Our patient showed substantial improvement of symptoms after joint treatment with antipsychotics and anti-SLE drugs. Similarly, the case report by Khalid et al. [18] showed favorable evolution after treatment with oral olanzapine (5 mg per day) to manage the lupus cerebritis flare-up following COVID-19 infection. It is highly recommended that patients presenting with psychosis features subsequent to covid-19 infection/ vaccination undergo close monitoring and immunological screening to promptly rule out an induced autoimmune disorder.

## Conclusion

We report an unusual case of new-onset acute psychosis as a manifestation of lupus cerebritis after concomitant COVID-19 infection and vaccination in a previously healthy young female. In light of the temporal link, we suggest the combined effect of both COVID-19 infection and mRNA vaccine could explain the development of SLE in our patient. In light of the reported case, we highly recommend a routine COVID-19 testing before vaccination in individuals with specific predisposition. However, the present conclusions need to be considered with cautious, and additional data and research is still needed before any firm conclusions about a causal link between COVID-19 mRNA vaccines and autoimmunity can be drawn.

## Data Availability

The datasets generated during the current study are not publicly available due to restrictions from the ethics committee but are available from the corresponding author on reasonable request.
